# Effects of miR-193a and sorafenib on hepatocellular carcinoma cells

**DOI:** 10.1186/1476-4598-12-162

**Published:** 2013-12-13

**Authors:** Alessandro Salvi, Isabel Conde, Edoardo Abeni, Bruna Arici, Ilaria Grossi, Claudia Specchia, Nazario Portolani, Sergio Barlati, Giuseppina De Petro

**Affiliations:** 1 Division of Biology and Genetics, Department of Molecular and Translational Medicine, University of Brescia, Viale Europa n. 11, 25123 Brescia, Italy; 2 Biostatistics Unit, Department of Molecular and Translational Medicine, University of Brescia, Brescia 25123, Italy; 3 Department of Clinic and Experimental Sciences, Surgical Clinic, University of Brescia, Brescia 25123, Italy

**Keywords:** miR-193a, Urokinase, Sorafenib, HCC

## Abstract

**Background:**

Hepatocellular carcinoma (HCC) is a challenging malignancy of global importance, it is the third most common cause of cancer-related mortality worldwide. In the last years the multikinase inhibitor sorafenib has been used for advanced HCC, but some patients do not benefit from this therapy; thus, novel therapeutic options based on molecular approaches are urgently needed. microRNAs are short non coding RNAs involved in several physiological and pathological conditions including HCC and increasing evidence describes miRs as good tools for the molecular targeted therapies in HCC. The purpose of this study was to identify novel approaches to sensitize the HCC cells to sorafenib by microRNAs targeting urokinase-type plasminogen activator (uPA).

**Methods:**

The miR-193a was validated as negative regulator of urokinase-type plasminogen activator (uPA) in 2 HCC undifferentiated cell lines by transient transfection of miR and anti-miR molecules. The molecular interaction between miR-193a and uPA mRNA target was verified by luciferase reporter assay. The miR-193a expression level was evaluated by stem-loop real time PCR in tumoral tissues from 39 HCC patients. The HCC cells were co-treated with sorafenib and miR-193a and the effects on cellular proliferation, apoptosis were tested. The effect of sorafenib on c-met expression levels was assessed by western blotting.

**Results:**

The miR-193a has resulted a negative regulator of uPA in both the HCC cell lines tested. The miR-193a expression has resulted dysregulated in tumoral tissues from 39 HCC patients. We found miR-193a down-regulation in HCC respect to peritumoral (PT) tissues and more in the cirrhotic HCCs than in non-cirrhotic ones. Transfection of HA22T/VGH HCC cells with miR-193a decreased proliferation and increased apoptosis, and combined treatment with miR-193a and sorafenib led to further proliferation inhibition.

**Conclusions:**

Our results present new advances in the post-transcriptional miR-mediated mechanisms of uPA and they suggest a new strategy to impair the aggressive behavior of HCC cells. Our findings could be helpful to explore novel approaches for multi-target and multi-agent therapies of the HCC.

## Background

Hepatocellular carcinoma (HCC) is one of the most lethal malignancies, it is the third most common cause of cancer-related mortality worldwide. Surgical resection and liver transplantation are first-line curative options for patients with early stage HCC, as they confer 5-year survival rates of 70%. Locoregional therapies such as transarterial chemoembolization and radiofrequency ablation are care for patients not suitable for surgery [1-3]. In recent years the multikinase inhibitor sorafenib has been used to treat advanced HCCs improving the overall survival of HCC patients from 7.9 months to 10.7 months and it is the sole systemic drug that is proved to be effective in this disease [4,5]. For this reason, efforts that focus on the implementation of personalized medicine approaches in HCC in the next years will be a challenge. It is well known that microRNAs (miRs) control a wide range of physiological and pathological processes, including cancer [6]. Dysregulation of miRs may play a relevant role in hepatocarcinogenesis and HCC progression [7]. For example, the hepatospecific miR-122 is significantly downregulated in more than 50-70% of HCCs and this loss of miR-122 expression is correlated with poor prognosis and metastasis of liver cancer [8]. In contrast, miR-21, miR-221 and miR-224 are generally reported to be upregulated in HCC tissues [9-11]. Several studies indicate that miRNAs expression may have clinical relevance as biomarkers for HCC stratification, early diagnosis or the follow-up of patients [12]. Additionally, studies showing that miRNAs themselves or anti-miRNA oligonucleotides can be successfully used for *in vitro* and *in vivo* modulation of miRNA actions have indicated significant potentials for molecular targeted therapy [13,14]. Additional studies have shown that some miRs may sensitize or improve the effects of the more conventional therapies in HCC cells. For example, an miR-122 mimetic alone or in combination with sorafenib reduced the tumourigenic properties of HCC cells and may therefore be a promising therapeutic regimen for liver cancer [15]. Chemoresistance to cisplatin is a major limitation of cisplatin-based chemotherapy in the clinic. In HCC patients treated with cisplatin-based chemotherapy, miR-199a-5p levels were significantly reduced; forced expression of miR-199a-5p promoted the cisplatin-induced inhibition of cell proliferation [16]. The resistance of HCC cells to 5-FU is mediated by miR-193a-3p *via* inhibition of the expression of serine/arginine-rich splicing factor 2 (SRSF2) expression. In turn, SRSF2 preferentially up-regulates the proapoptotic splicing form of caspase 2 (CASP2L) and sensitizes HCC cells to 5-FU. Forced changes of miR-193a-3p level were shown to reverse the 5-FU sensitivity, in cell culture and in *nude* mice [17]. It is well known that the serine protease urokinase type plasminogen activator (uPA) is a responsive therapeutic target for HCC and others malignancies and its overexpression correlates with tumour invasion and metastasis [18-22]. In this work, to study the co-treatment of HCC cells with sorafenib and miRNAs targeting uPA we have first validated the miR-193a-3p as a negative regulator of uPA in HCC cells; furthermore, we have tested the effects of miR-193a-3p in combination with sorafenib.

## Results

### miR-193a negatively regulates uPA expression in HCC derived cell lines

**Figure 1:**
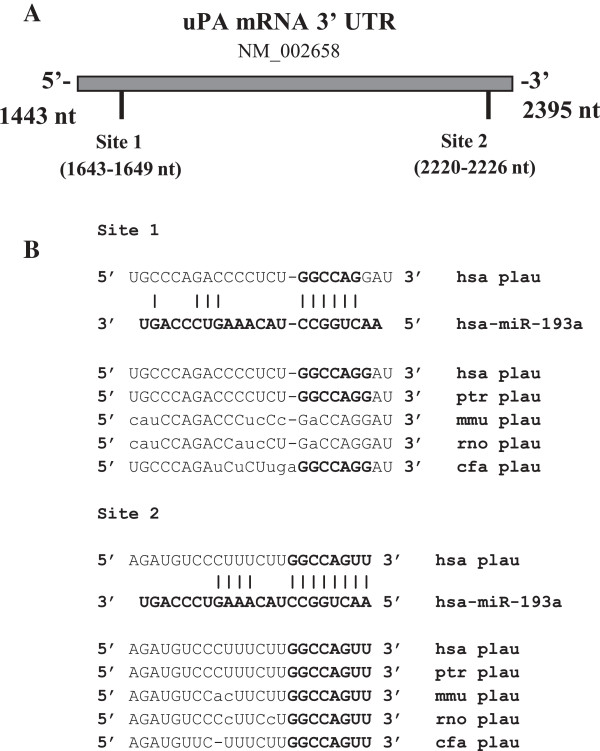
**uPA putative binding sites for miR-193a located in the 3′-UTR mRNA and its phylogenetically conservation. (A)** 3′-UTR uPA mRNA displays two putative binding sites for miR-193a. **(B)** The complementarity between miR-193a and the putative uPA 3′-UTR target sites are shown. The conserved bases of the putative miR-193a target sequence are also shown. hsa, *Homo sapiens*; ptr, *P. troglodytes* (chimpanzee); mmu, *M. musculus* (mouse); rno, *R. norvegicus* (rat); cfa, *C. familiaris* (dog).

Before studying the co-treatment of the HCC cells with sorafenib and miRNAs, we studied miRs that were predicted by bioinformatic tools to putatively regulate uPA expression. We have previously predicted miR-193a to be a negative regulator of uPA expression [23], among others. There are two putative binding sites located at the 3′UTR uPA mRNA (Figure 1A). Both sites, but in particular site 2 (nt 2220–2226 NCBI access number NM_002658), are phylogenetically conserved across the species (Figure 1B).

**Figure 2:**
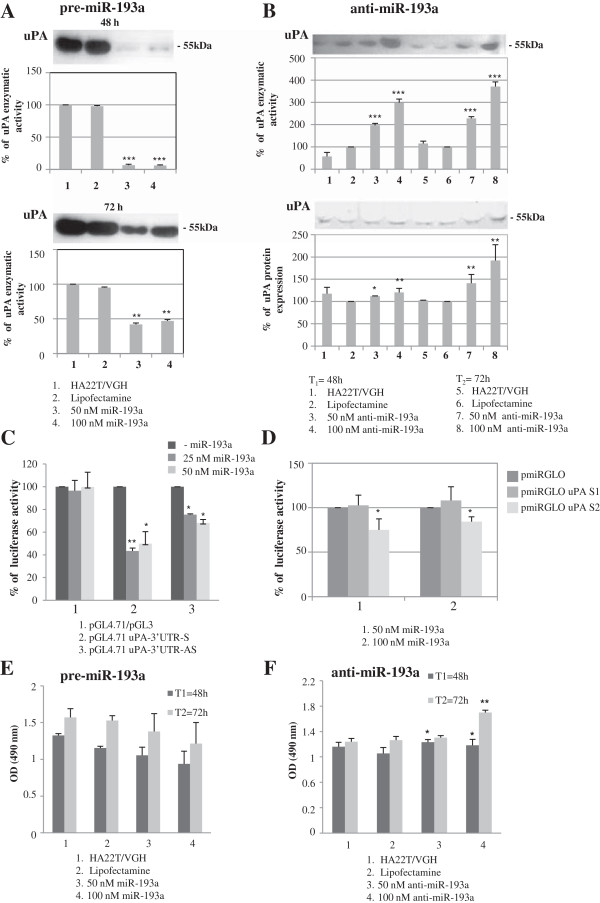
**Experimental validation of miR-193a as negative regulator of uPA in HA22T/VGH cells. (A)** Enzymatic activity evaluated by zymography after pre-miR-193a transfection in HA22T/VGH cells. Three experiments were performed; histograms represent the mean values, bars are the SE. ^*^p < 0.05; ^**^p < 0.01 ^***^p < 0.001 *versus* Lipofectamine in a t-Test analysis for unpaired comparison. **(B)** anti-miR-193a transfection in HA22T/VGH cells causes increase of uPA protein expression and its correspondent enzymatic activity. Three experiments were performed; histograms represent the mean values, bars are the SE. ^*^p < 0.05; ^**^p < 0.01 ^***^p < 0.001 *versus* Lipofectamine in a t-Test analysis for unpaired comparison. **(C-F)** Dual-luciferase reporter assay and cellular proliferation. **(C)** The 3′-UTR of uPA was cloned in the pGL4.71 reporter plasmid after the *Renilla* luciferase CDS in sense (pGL4.71 uPA-3′ UTR-S) and antisense (pGL4.71 uPA-3′ UTR-AS) orientation. The constructs were cotransfected into HA22T/VGH cells with 0, 25 and 50 nM miR-193a mimics. Luciferase activity was normalized relative to a simultaneosly transfected firefly luciferase expression plasmid (pGL3). The construct pGL4.71 uPA-3′ UTR-S determined the inhibition of the luciferase activity, ^*^p < 0.05; ^**^p < 0.01 *versus* –miR-193a. **(D)** 30 nt-sequences containing the putative binding site 1 and 2 were cloned after the firefly luciferase CDS of the pmiRGLO plasmids carrying also the Renilla luciferse gene. The 2 plasmids (pmiRGLO uPA S1, pmiRGLO uPA S2) were cotrasfected with 50 nM and 100 nM miR-193a mimics in HA22T/VGH cells. Site 2 resulted in the inhibition of the luciferase activity while site 1 did not, ^*^ p < 0.05 *versus* pmiRGLO. **(E-F)** The transfection of pre-miR-193a and anti-miR-193a in HA22T/VGH cells led to a low level of proliferation inhibition and induction respectively. The histograms represent the mean of three experiments; bars are the SE. ^*^p < 0.05; ^**^p < 0.01 *versus* HA22T/VGH.

**Figure 3:**
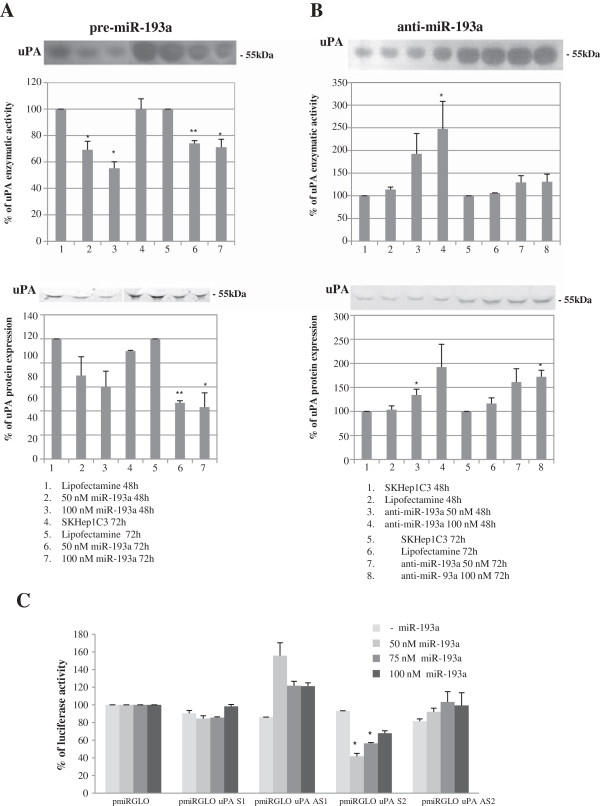
**Experimental validation of miR-193a as negative regulator of uPA in SKHep1C3 cells. (A)** pre-miR-193a transfections in SKHep1C3 cells inhibited uPA protein expression and the enzymatic activity. **(B)** Anti-miR-193a transfection in SKHep1C3 cells led to up-regulation of uPA protein expression and the enzymatic activity up-regulation, ^*^p < 0.05; ^**^p < 0.01 *versus* Lipofectamine in a t-Test analysis for unpaired comparison. **(C)** The dual luciferase assay shows that the site 2 was directly bound by miR-193a but the site 1 was not. pmiRGLO uPA S1 and pmiRGLO uPA S2 are the luciferase constructs expressing the predicted 3′ UTR mRNA uPA binding site 1 and site 2. The pmiRGLO uPA AS1 and pmiRGLO uPA AS2 are the constructs expressing the corresponding control antisense sequences, ^*^p < 0.05 versus – miR-193a.

We transfected the HCC-derived cell line HA22T/VGH with pre-miR-193a molecules and we found that the uPA enzymatic activity was significantly inhibited in the transfected cells compared with control cells (Figure 2A). Conversely, the transfection of anti-miR-193a molecules resulted in upregulation of uPA enzymatic activity and protein expression, 48 h and 72 h after transfection (Figure 2B). To determine whether miR-193a could directly interact with 3′ UTR uPA mRNA we performed the luciferase reporter assays. The entire 3′UTR uPA mRNA cloned downstream to the luciferase CDS resulted in inhibition of luciferase activity when the construct (pGL4.71 uPA-3′ UTR S) was co-transfected with pre-miR-193a (Figure 2C histogram 2). As shown in Figure 2D the predicted binding site 2, cloned in another type of luciferase plasmid (pmiRGLO uPA S2), was directly recognized by miR-193a while the site 1 (pmiRGLO uPA S1) was not. To understand whether the miR-193a may influence the malignant phenotype of the HA22T/VGH cells we transfected the cells with pre-miR-193a or anti-miR-193a and we assessed their effects on cellular proliferation. We observed a low decrease in cell proliferation when transfecting pre-miR-193a molecules (up to 21% at 72 h from transfection at 100 nM concentration, Figure 2E) however we obtained an induction of proliferation when transfecting anti-miR-193a molecules (up to 23% at 72 h from transfection at 100 nM concentration; p < 0.01; Figure 2F). The validation of miR-193a as negative regulator of uPA was extended to the HCC cell line SKHep1C3. The transfection of pre-miR-193a resulted in downregulation of uPA protein/enzymatic activity (Figure 3A), while transfection of anti-miR-193a (Figure 3B) up-regulated the level and activity of uPA. As determined in the luciferase reporter assay (Figure 3C), site 2 was directly bound by miR-193a whereas site 1 was not recognized by miR-193a, as observed in the HA22T/VGH cells.

### miR-193a is downregulated in HCC biopsy specimens

**Figure 4:**
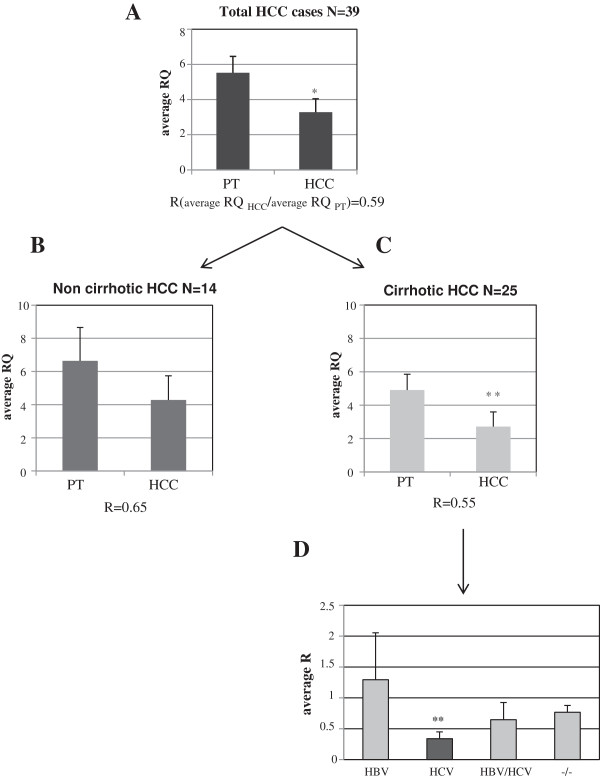
**miR-193a expression analysis by Real-Time PCR in tumoral tissues from biopsy samples of HCC patients. (A)** The level of mature miR-193a is generally down-regulated in HCC compared with the corresponding PT tissues average RQ_PT_ = 5.53 ± 0.93, RQ_HCC_ = 3.27 ± 0.77; p < 0.05. **(B)** miR-193a expression level in HCCs developed in non-cirrhotic and **(C)** cirrhotic liver. **(D)** Cirrhotic HCCs stratified on the basis of the type of hepatitis viral infections. The class of HCV showed the lowest average R (average R = 0.338; p < 0.01 in a t-Test analysis for single group mean with expected value = 1).

The expression levels of mature miR-193a were assessed by real time PCR. miR-193a resulted down-regulated in HCC tissues from biopsy specimens of 39 HCC patients (Additional file 1) with respect to their peritumoral (PT) counterparts (average RQ_PT_ = 5.53 ± 0.93; average RQ_HCC_ = 3.27 ± 0.77 p < 0.05; R = (RQ_HCC_/RQ_PT_) 0.59) (RQ = relative quantification) (Figure 4A and Additional file 2 and Additional file 3). We have stratified the cases on the basis of presence or absence of cirrhosis as background liver disease; for the class of non-cirrhotic HCCs (n = 14) we observed an average RQ_PT_ = 6.6 ± 2, an average RQ_HCC_ = 4.3 ± 1.46 with a ratio (R = RQ_HCC_/RQ_PT_) value of 0.65 (Figure 4B and Additional file 3 and Additional file 4); and for the class of cirrhotic HCCs (n = 25), the average RQ_PT_ was 4.9 ± 0.94; the average RQ_HCC_ was 2.7 ± 0.88 p < 0.01 with an R value of 0.55 (Figure 4C and Additional files 3 and Additional file 4). We further stratified the cirrhotic HCCs on the basis of the type of hepatitis virus infections and for each sub-class we calculated the average R (RQ_HCCn_/RQ_PTn_). We found that the class of HCV (n = 10) presented the lowest average R (R = 0.338) which was significantly different from the expected value = 1, p < 0.01; the R values of the HBV (n = 4), HBV + HCV (n = 4) and −/− (n = 4) classes were 1.29 ± 0.75; 0.645 ± 0.28 and 0.77 ± 0.11 respectively and they did not significantly differ from 1 (Figure 4D). By stratifying the non-cirrhotic HCCs on the basis of the type of hepatitis virus infection we have found no expression variation (Additional file 5). Interestingly, when we considered all the HCV patients (n = 15) with or without cirrhosis the mean R value was 0.604 ± 0,14 which was significantly different from the expected value = 1, p = 0.0167 (Additional file 5).

### Effects of miR-193a ectopic expression and sorafenib on the HCC cells

**Figure 5:**
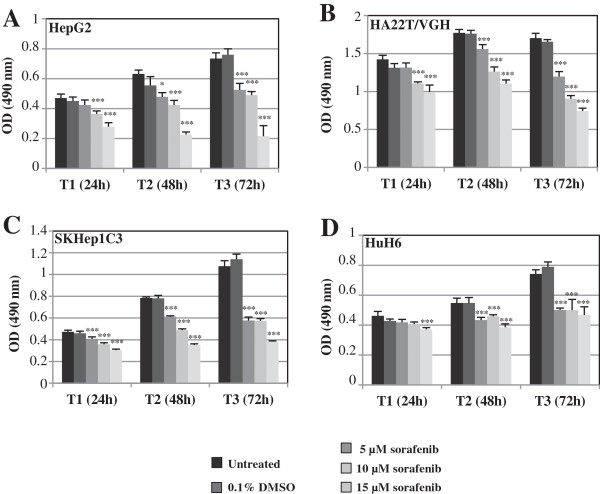
**Effects of the sorafenib treatment on HCC cell lines.** The HepG2, HA22T/VGH, SKHep1C3 and HuH6 cell lines were treated with sorafenib (5, 10, 15 μM concentration) for 3 days (24, 48 and 72 h). The proliferation was inhibited in a dose-dependent manner in all the cell lines considered **(A-D)**. The most responsive HCC cell line was HepG2 (the highest percentage of the inhibition of proliferation was 72% with 15 μM of sorafenib at T = 72 h) **(A)**. Statistical significance was determined by one way ANOVA with Bonferroni correction ^*^p < 0.05; ^***^p < 0.001.

**Figure 6:**
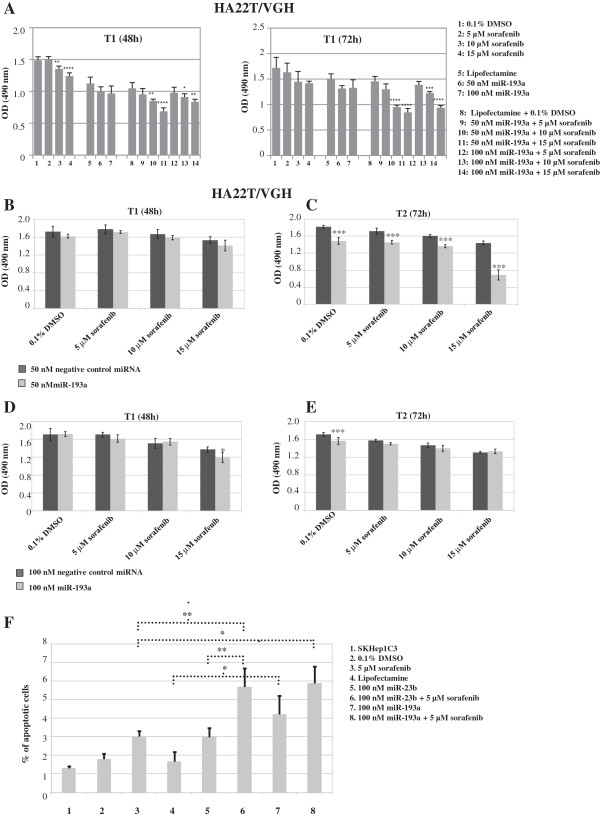
**Effects on cellular proliferation and apoptosis of combined treatment with sorafenib and miR-193a/miR-23b in HCC cell lines. (A)** The MTT assay showed that the growth of the HA22T/VGH cells was significantly reduced after the combined treatments of sorafenib *plus* miR-193a. ^*^p < 0.05; ^**^p < 0.01, ^***^p < 0.001, ^****^p < 0.0001 in a one-way ANOVA followed by Bonferroni correction: for sorafenib alone *versus* 0.1% DMSO, for miR alone *versus* Lipofectamine, for the co-treatments *versus* Lipofectamine plus 0.1% DMSO. **(B-E)** The HA22T/VGH cells were transfected with negative control miRNA or miR-193a and treated with either vehicle (0.1% DMSO) or different concentrations of sorafenib (5-10-15 μM). Two-way ANOVA followed by Bonferroni correction was used to establish whether significant difference existed between negative control miRNA transfected cells and miR-193a transfected cells; ^***^p < 0.001. **(F)** TUNEL assay in SKHep1C3 cells. The cells transfected with 100 nM miR-23b or miR-193a and then treated with 5 μM sorafenib showed increased percentages of apoptotic cells (up to 1.95 times those of cells treated with sorafenib alone). ^*^p < 0.05; ^**^p < 0.01 in a two-way ANOVA followed by Bonferroni correction.

To study the effects of the co-treatment on the HCC cells with miR-193a and sorafenib we have first of all evaluated the effect of sorafenib on cellular proliferation. The treatment of 4 HCC cell lines with sorafenib (5, 10, 15 μM concentration) for 3 days (24, 48 and 72 h) inhibited proliferation (Figure 5A-D). The most sensitive HCC cell line was HepG2 which had the highest percentage of inhibition of proliferation (72%) 72 h following treatment with 15 μM of sorafenib (Figure 5A). It is known that some microRNAs can improve the sensitivity of cancer cells to conventional drugs and chemotherapeutic agents, for this reason we tested whether miR-193a could increase the effect of sorafenib on HCC cells. We treated HA22T/VGH ectopically expressing miR-193a with sorafenib and monitored cell growth. The MTT assay data showed that the growth of the HA22T/VGH cells was significantly reduced upon the combined treatments of miR-193a and sorafenib (Figure 6A, B-E ). The fold change increases (calculated by dividing the percentage of proliferation inhibition of sorafenib + miR-193a cells and the percentage of proliferation inhibition of the cells treated with sorafenib alone) were between 2.3 and 2.6 both at 48 h and 72 h after transfection respectively (Figure 6A) and 2.1 in the cotreated cells with 50 nM miR-193a and 15 μM sorafenib *vs* 50 nM negative control miRNA and 15 μM sorafenib (Figure 6C).

The quantification of TUNEL-positive SKHep1C3 cells showed that miR-193a overexpression can induce HCC cell apoptosis (Figure 6F, histogram n. 7), that transfection with 100 nM miR-23b or miR-193a and treatment with 5 μM sorafenib increased the number of apoptotic cells up to 1.89 and 1.95 fold respectively compared with treatment with sorafenib alone (Figure 6F, histograms 6 and 8) and that the combined treatment of miR-23b and sorafenib increased the number of apoptotic cells compared with treatment with miR-23b alone. We chose also miR-23b for this analysis because we previously reported that miR-23b is a negative regulator of uPA and c-met in SKHep1C3 cells and its ectopic expression negatively regulates properties related to cellular aggressiveness [23].

### Sorafenib mediates c-met expression downregulation

**Figure 7:**
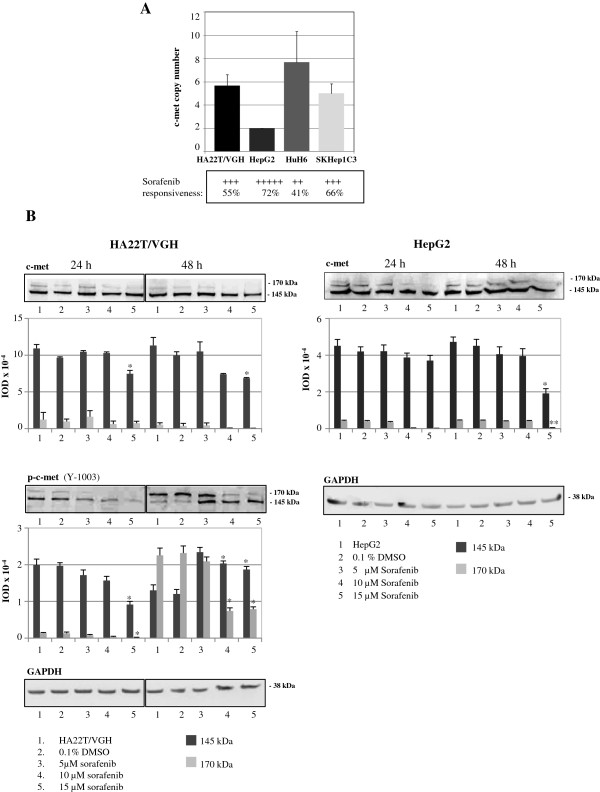
**Effects of sorafenib on c-met protein expression levels. (A)** c-met copy number analysis in the 4 HCC cell lines considered and comparison with the highest percentage of inhibition of proliferation observed after sorafenib treatments. **(B)** Western blot and densitometric analysis of c-met, p-c-met (Y1003) and GAPDH in the HA22T/VGH and HepG2 cells treated with sorafenib ^*^p < 0.05; ^**^p < 0.01 *versus* HA22T/VGH or HepG2 in a t-Test analysis for unpaired comparison.

To determine the relationship between the RTK c-met copy number and the cellular proliferation after sorafenib treatment, the c-met copy number was calculated in the four HCC cell lines considered. Interestingly, there was an inverse trend between the highest percentage of obtained inhibition of proliferation after sorafenib treatment and the c-met copy number (Figure 7A). The HA22T/VGH cell line that displayed an intermediate sensitivity to sorafenib and the most sensitive HepG2 cells were analyzed for c-met protein expression.

The tyrosine kinase c-met is synthesized as a 170-kDa precursor protein that is further cleaved to form an α-chain of 50 kDa linked by disulfide bonds with a 145-kDa β-chain. In the HA22T/VGH and in the HepG2 cells treated with sorafenib, the c-met precursor of 170 kDa resulted inhibited mainly after treatment with 10 and 15 μM of sorafenib at both 24 h and 48 h time points and the c-met β chain of 145 kDa decreased mainly at 15 μM sorafenib at the later time point (48 h) (Figure 7B). The levels of p-c-met (Y1003) in HA22T/VGH cells were inhibited at the 24 h time point both the 170 kDa precursor protein and the 145-kDa β-chain; this could reflect the c-met protein expression level. At T = 48 h we have found a decrease of the precursor form of 170 kDa of p-c-met after the treatment with 10 and 15 μM of sorafenib respect to control and 5 μM dose. We have also detected a higher amount of the 145 kDa form of p-c-met in the sorafenib treated cells compared with the untreated cells. It is known that the phosphorylation at the Y1003 plays a role in the ubiquitination of the c-met and thus in its degradation [23]. All together these observations indicate that the sorafenib might mediate the degradation of the c-met by favoring the ubiquitination and thus its degradation.

## Discussion

It is well known that the uPA and the RTK c-met are generally overexpressed in HCC. They are considered negative prognostic factors and responsive therapeutic targets for this type of cancer [20,24,25]. We have previously shown that miR-23b targets both uPA and c-met expression in HCC cell lines and the ectopic overexpression of miR-23b reduces the malignant properties of the cells [23]. Here, with the aim to increase the molecular tools available to silence uPA we have studied the hsa-miR-193a-3p previously predicted by us to target uPA [23]. Our results clearly show that miR-193a negatively regulates uPA in 2 HCC-derived cell lines. Other authors have previously described uPA as a target of miR-193a-3p in breast-cancer cell lines [26,27]. It is known that a given miR may control the expression level of a gene in some biological context (i.e. cells, tissues) but not in others; therefore the experimental validation of this miR in different cell lines is necessary. Because uPA levels are generally higher in HCC tissues with respect to their adjacent non-tumoural counterparts, we quantified the miR-193a expression in tissues from biopsy specimens of donor patients. In agreement with the hypothesis that miR-193a inhibits uPA expression, mature miR-193a generally resulted down-regulated in HCC tissues compared with the PT counterparts (R = 0.59; p < 0.05). These novel results support the *idea* that miR-193a would target uPA not only in HCC cell lines, but also in human liver tissue making miR-193a a promising tool for down-regulating uPA expression levels. Interestingly, when considering the critical importance of the presence or absence of hepatic cirrhosis for the classification and the subdivision of the patients, we observed a lower and more statistically significant level of miR-193a in cirrhotic HCCs (R = 0.55; p < 0.01) compared with those that were non-cirrhotic (R = 0.65). Furthermore, cirrhotic HCCs with HCV infection showed a very low miR-193a expression level compared with PT tissues (R = 0.338), but HBV, HBV + HCV and −/− did not display any substantial miR-193a expression changes. We are aware that the classification of the cirrhotic HCCs on the basis of the type of hepatitis virus infection is made on small sample size; thus this analysis will be extended to a larger number of HCC cases. It is known that miRs can alter their expression as a result of viral infection or in particular pathological and stress conditions [28]. Notably, hepatic cirrhosis decreased the expression level of miR-193a (RQ_PTnc_ = 6.6; RQ_PTc_ = 4.9), a further decrease in miR-193a levels was observed as a result of hepatocyte transformation (RQ_HCCnc_ = 4.3; RQ_HCCc_ = 2.7). It is not uncommon that miRs can vary during the different stages from liver healthy tissues to pathological hepatic lesions that often precede the onset of HCC. In a previous report we found that miR-24, miR-27a and miR-21 were differentially expressed in cirrhotic/non-cirrhotic HCCs [29]. Therefore, to hypothesize a putative role of miR-193a as marker of stage progression it will be necessary to evaluate its expression also in the condition of healthy and unhealthy liver tissues (i.e., chronic hepatitis, steatosis).

It is a shared opinion that novel therapies for HCC based also on molecularly targeted-therapy are urgently needed. The sorafenib is an oral multikinase inhibitor that targets Raf, VEGFR-2/3, PDGFR-β, Flt3 and c-kit. It is used to treat the advanced HCC, but some patients do not benefit from this therapy [5]. One of the main problems is that cancer acquires resistance to kinase inhibitors because of genetic modifications or activation of alternative pathways [30]. An effective method to sensitize the cancer cells to sorafenib or the use of combined therapies are ambitious objectives to pursue. In fact, miR-193a transfection decreased proliferation and increased apoptosis and combined treatment of HCC cells with miR-193a and sorafenib showed additional effects in terms of cellular proliferation inhibition. The data obtained from the c-met copy number assay indicate an inverse trend between the number of c-met copies and the degree of reduced proliferation obtained following sorafenib treatment in the four HCC cell lines. It is known that c-met amplification negatively affects the survival of surgical resected non-small cell lung patients and the c-met gene copy number was linked to resistance to the tyrosine kinase inhibitor gefitinib in non-small cell lung cancer patients [31,32]. The fact that c-met copy number (and reasonably the c-met expression level) may have a role in the efficacy of sorafenib, at least *in vitro*, led us to analyze the expression level of c-met protein following sorafenib treatments in cells. The c-met protein levels were inhibited in treated HA22T/VGH and HepG2 cells and this may indicate, for the first time in the present work, a direct or an indirect role of sorafenib in controlling c-met expression. We further observed that the amount of the phosphorylated form (Y1003) of the c-met β chain of 145 kDa was increased in the treated HA22T/VGH cells at 48 h time point following treatment. The tyrosine residue (Y1003) located in the juxtamembrane domain, upon phosphorylation, binds to the E3-ubiquitin ligase Cbl, which promotes receptor ubiquitination, endocytosis and degradation [33]. We therefore surmise that sorafenib may decrease the expression of c-met by promoting its degradation at least at the later time points following the treatment, and this could help in understanding an aspect of the molecular mechanisms of sorafenib which have not been fully elucidated. A recent study indicates that sorafenib significantly altered expression levels of 826 and 2011 transcripts in HepG2 and Huh7 cells respectively [34], indicating the complexity of the mechanism of action of sorafenib. Further studies on this topic are necessary to make more effective the use of sorafenib as anti-cancer drug.

## Conclusions

Our characterization of the down-regulated profile of miR-193a in HCC might be helpful to differentiate molecular subtypes of human hepatocellular carcinoma by matching the miR-193a expression with some clinical features of patients. Furthermore, our findings may shed light in defining a pre-clinical therapeutic schedule for HCC based on the use of miR-193a and miR-23b given alone or in combination with sorafenib. Our preliminary observations on the role of sorafenib in mediating, directly or indirectly, the down-modulation of c-met expression prompt further studies to acquire new knowledge on the molecular mechanism of action of this drug.

## Methods

### Cell culture and treatments

SKHep1Clone3 (SKHep1C3), selected from human HCC-derived cells (SKHep1: ATCC HTB-52), was maintained in Earle’s MEM (Life Technologies, Carlsbad, CA, USA) supplemented with 10% foetal bovine serum (Life Technologies) at 37°C in a 5% CO_2_ incubator. Differentiated human HCC-derived cells (HepG2, ATTC HB-8065; HuH-6) and HA22T⁄VGH undifferentiated HCC-derived cells were maintained in RPMI-1640 (Life Technologies) supplemented with 10% foetal bovine serum at 37°C in a 5% CO_2_ incubator. The HuH-6 and HA22T/VGH cells were kindly provided by N. D’Alessandro (University of Palermo, Italy). Sorafenib [N-(3-trifluoromethyl-4-chlorophenyl)-N-(4-(2-methylcarbamoylpyridin-4-yl) oxyphenyl) urea] was synthesized at Bayer Corporation (West Haven, CT). This compound was dissolved in 100% DMSO (Sigma, St. Louis, MO) and diluted with DMEM or MEM to the desired concentration; a final DMSO concentration of 0.1% was used for *in vitro* studies. DMSO was added to cultures at 0.1% (v/v) as a solvent control.

### Transient transfection of HA22T/VGH and SKHep1C3 with miR-193a

Molecules of double-stranded RNAs that mimic endogenous hsa-miR-193a mature miR (5′-AACUGGCCUACAAAGUCCCAGU3′), anti-miR-193a were purchased from Life Technologies. For the experimental validation of miR-193a as negative uPA regulator, HA22T/VGH and SKHep1C3 cells were seeded in complete medium at 80% confluence in a 24-well plate (80,000 cells/well). Then, 24 h after seeding, the cells were transfected into serum-free RPMI or Earle’s MEM, respectively, with 50 and 100 nM of pre-miR-193a and/or anti-miR-193a using Lipofectamine transfection reagent, according to the manufacturer’s instruction (Life Technologies). The transfection medium was replaced with the complete medium after 24 h. The conditioned media and cell lisates were collected 48 h and 72 h after transfection and quantified for zymography and western blot analysis.

### Western blot and zymography

The media for uPA expression analysis were collected from cultures of both nontransfected and transfected cells. Constant amounts of proteins were loaded (Additional file 6), under non-reducing conditions, on a Novex NuPAGE (4–12%) Bis/Tris gel (Invitrogen), or on an 8% SDS polyacrylamide gel, which was blotted onto a nitrocellulose membrane (NM). NMs were immunoreacted using rabbit anti-human uPA (1:1000 in 1% BSA) and alkaline phosphatase-conjugated anti-rabbit IgG (1:7500 in 0.3% BSA); for zymography, NMs were overlayed onto casein agar containing 2 μg/mL human plasminogen (Technoclone GmbH, Vienna, Austria) to evaluate uPA activity. To evaluate c-met and GAPDH expression in the HA22T/VGH untreated and treated cells with 5-10-15 μM sorafenib, the cell extracts were collected from 24 h and 48 h cultures by adding 0.05% SDS. Constant amounts of proteins were loaded, under reducing conditions, on a Novex NuPAGE (4–12%) Bis/Tris gel or on an 8% SDS polyacrylamide gel, and were then transferred to NMs. The blots were immunoreacted using rabbit anti-human c-met (1:1000 in 0.3% BSA) and rabbit anti-human p-c-met, (Y1003) (1:1000 in 0.3% BSA), alkaline phosphatase-conjugated anti-rabbit IgG secondary antibody (1:7500 in 0.3% BSA), or mouse monoclonal antibodies anti-GAPDH (1:300 in 1% BSA) and alkaline phosphatase-conjugated anti-mouse IgG secondary antibody (1:7500 in 0.3% BSA). The results of the immunoreaction were detected with Nitroblue tetrazolium and bromochloroindolyl phosphate (Promega). The bands corresponding to c-met (170 and 145 kDa), uPA and GAPDH were scanned and analyzed using a digital system (Gel-Pro Analyzer), and the integrated optical density (IOD) values were expressed in pixels.

### Cell proliferation assays

The cellular proliferation was analysed using the CellTiter 96 Aqueous One Solution reagent (Promega, San Diego, CA, USA) after the treatment with sorafenib and/or pre-miR-193a, pre-miR precursor negative control #1 (Life Technologies) and anti-miR-193a transfections. The cells were seeded in 96-well plates (5 replicates for each experimental condition) at a density of 4 × 10^3^ cells/well in a complete cultured medium and 15 μl/well of sterile CellTiter reagent was added at the established time after transfection and/or sorafenib treatments. The plates were measured 2 h after CellTiter addition using a microplate reader. The absorbance values at 490 nm were directly proportional to the number of living cells in the culture.

### In situ cell death

The effects of sorafenib on apoptosis in miR-193a- and miR-23b [23] transfected or non transfected HCC cells were measured using the TUNEL assay (terminal deoxynucleotidyl transferase dUTP nick end labeling; Roche Molecular Biochemicals). SKHep1C3 cells were seeded on 13 mm-diameter glass coverslips in 24-well plates (30,000cells/well) and after 24 h the cells were transfected with 100 nM miR-193a or 100 nM miR-23b; after 24 h the media were replaced and 5 µM sorafenib was added for 24 h. Cell death was detected *in situ* by enzymatic labelling of DNA strand breaks using TUNEL, according to the manufacturer’s instructions. Briefly, the DNA end-labelling reaction was performed using terminal deoxynucleotidyl transferase (TdT) and tetramethylrhodamined UTP (TMR-dUTP), followed by direct analysis of fluorescent cells. Positive controls were obtained by treating cells with 7 U/ml DNase for 10 min at room temperature. Then, the nuclei of the cells were counterstained with 4′, 6-Diamidino-2-Phenylindole (DAPI): the samples were then analyzed on a fluorescence microscope under 20× magnification. The percentage of TUNEL-immunostained nuclei (TUNEL labelling index) was calculated in each sample using the formula: number of labelled nuclei/total number (labelled + unlabelled) of nuclei × 100. Measurements were carried out using ImageJ 1.45S software. This program allows the user to count random fields (4 random fields were photographed for each sample and 4 measurements were taken for each picture both for TMR and DAPI staining to ensure that the percentage of apoptotic cells are representative of the entire sample).

### Luciferase reporter assays

The human 3′ untranslated region (3′UTR) uPA mRNA (937 bp) were PCR-amplified from cDNA of SKHep1C3 cells, using primers containing flanking *Xba*I recognition sequences (Fw: 5′-GC**TCTAGA**CTGAGGGTCCCCAGGGAG-3′; Rev: 5′-GC**TCTAGA**TTCATCAGAAAAATCACATTTTATTG -3′). PCRs were performed using PFU Taq polymerase (Promega, San Diego, CA, USA) with proofreading activity. The PCR products were ligated in the *Xba*I restriction site downstream of the *Renilla* luciferase coding region of the pGL4.71 vector (Promega), in which the simian virus 40 promoter region from the pGL3-Promoter vector (Promega) had been previously cloned to obtain the pGL4.71P plasmid. The correct orientation of the insert was verified by sequencing. HA22T/VGH cells were seeded at a confluency of 60–80%; 24 h after seeding the cells were transfected with 0-25-50 nm pre-miR-193a and were then transfected with the luciferase reporter constructs (0.5 μg) 48 h after seeding using Lipofectamine 2000 transfection reagents according to the manufacturer’s instruction. Seventy-two hours after seeding, the cells were washed with NaCl/P_i_ and lysed with passive lysis buffer (Promega, San Diego, CA, USA), and the firefly luciferase (f-luc) and *Renilla* luciferase (r-luc) activities were determined using the dual-luciferase reporter assay system (Promega) and a luminometer. The relative reporter activity was obtained through normalization to the f-luc activity. To verify which putative binding site was recognized by miR-193a, two double-strand oligonucleotides containing flanking restriction sequences for the enzymes *XbaI* and *DraI* and the 2 putative binding sites were cloned into the pmiRGLO Dual-Luciferase miRNA Target expression vector. The sequences of the oligonucleotides were the following: for site 1: TOP-S1 5′-**AAA**GCCCAGACCCCTCTGGCCAGGATGGAGGGG**T**-3′; BOTTOM-S1 5′-**CTAGA**CCCCTCCATCCTGGCCAGAGGGGTCTGGGC**TTT**-3′; for site 2: TOP-S2 5′-**AAA**TCCCTTTCTTGGCCAGTTATCCCTTCCTTT**T**-3′, BOTTOM-S2 5′-**CTAGA**AAAGGAAGGGATAACTGGCCAAGAAAGGGA**TTT**-3′. The plasmid was first linearized with the restriction enzymes *XbaI* and *DraI* and the annealed oligonucleotides were cloned downstream to the firefly luciferase CDS. The plasmids expressing the site 1 and site 2 were named pmiRGLO uPA S1 and pmiRGLO uPA S2 respectively, and the control plasmids with the corresponding sequences cloned in antisense orientations were called pmiRGLO uPA AS1 and pmiRGLO uPA AS2. The empty plasmid was named pmiRGLO. Firefly luciferase activity was used as the primary reporter to monitor the regulation of miR-193a and *Renilla* luciferase acted as a control reporter for normalization. The constructs were co-transfected into HA22T/VGH and SKHep1C3 cells with 0, 50, 75, 100 nM pre-miR193a and the evaluation of luciferase activity was performed as decribed above.

### Tissues and clinicopathological features of HCC and real-time evaluation of mature miR-193a expression in tumoural and peri-tumoural (PT) human tissues

All human HCC samples (*n* = 39) as well as the corresponding PT non-tumour samples (resected 1–2 cm from the malignant tumour) were obtained from HCC patients for pathological examination. Each biopsy specimen was obtained with the patient’s informed consent under standard conditions of sampling and processing [23]. Each specimen was determined to be HCC or PT by pathological examination. In this study, 39 HCC subjects underwent surgical resection. The subjects consisted of 26 men and 13 women (38 Italian and 1 Chinese) ranging from 38 to 82 years of age (mean age: 67.8 ± 9.06 years). The subjects did not have any apparent distant metastases, and none had been previously treated for HCC. We have subdivided the cases on the basis of presence or absence of liver cirrhosis (25 HCC with cirrhosis, 14 HCC without cirrhosis); the patients were tested for the presence of the hepatitis B virus (HBV) and hepatitis C (HCV) virus. Fifteen patients were positive for HCV, 9 were positive for HBV, 4 were positive for both HBV and HCV, and 6 were negative for both HBV and HCV; for 5 patients no information was available (Additional file 1). The total RNA from tissue samples was isolated using TRIzol reagent (Invitrogen), according to the manufacturer’s instructions. To measure the amount of mature miR-193a, a two-step TaqMan real-time PCR analysis was performed, using primers and probes obtained from Life Technologies-Applied Biosystems. In a reaction volume of 15 μl, cDNA was synthesized from 50 ng of total RNA, using reverse transcriptase and the stem–loop primer for miR-193a (Applied Biosystems; PN 4427975) or RNU66 (internal control; Applied Biosystems; PN 4373382) contained in the TaqMan MicroRNA Reverse Transcription kit (Applied Biosystems, Foster, CA, USA). The reverse transcriptase reaction was performed by incubating the samples at 16°C for 30 min, 42°C for 30 min, and 85°C for 5 min. The PCR reaction (20 μL) contained 1.3 μL of reverse transcriptase product, 10 μL of Taq-Man 2× Universal PCR Master Mix, and 1 μL of the appropriate TaqMan MicroRNA Assay (20×) containing primers and probes for the miR of interest. The PCR mixtures were incubated at 95°C for 10 min, and this was followed by 40 cycles of 95°C for 15 s and 60°C for 60 s. PCR reactions were performed in triplicate using a 7500 real time PCR system. The expression of miR-193a was based on the ΔΔC_T_ method, using RNU66 as an internal control. For each case the ratio (R) between the relative levels in HCC and those in PT was assessed. The level of expression of the miRNAs was considered to be decreased for a R value <0.7 and increased for a R value >1.3. A value between 0.7 and 1.3 was defined as having no change in expression level [29].

### c-met copy number evaluation

DNA from HCC cell lines was extracted using TRizol reagent, according to the manufacturer’s instructions. Quadruplicates of each sample using 20 ng of genomic DNA *per* sample were amplified using four different TaqMan probes (Applied Biosystems, Life Technologies) spanning the entire c-met gene and chosen within the exon 2 (hs 01375065-cn), intron 5 (hs 04992567-cn), exon 8 (hs 02633538-cn) and exon 21 (hs 01932765-cn). The PCR mixtures were incubated at 95°C for 10 min and this was followed by 40 cycles at 95°C for 15 s and 60°C for 60 s. The method of relative quantification was used to determine the relative copy number of the c-met in each DNA sample, normalized to the known copy number of the reference gene RNase P. The RNase P probe was run together with each c-met probe using duplex real-time PCR (Applied Biosystem 7500).

### Statistical analysis

Each experiment was carried out at least twice. Histograms represent the mean values, and bars indicate standard errors (SE) of the mean. For the data shown in Figures 2, 3, 4 and 7 statistical analysis (Student’s t-test) was performed with kyplot, version 2.0 beta 13 (KyensLab Incorporated, Tokjo, Japan; www.kyenslab.comhttp://). For the data shown in Figures 5 and 6 statistical analysis (one way ANOVA with Bonferroni correction; two way ANOVA with Bonferroni correction) was performed with GraphPad Prism 6.0 (GraphPad Softwares Inc, San Diego, CA, USA). Data were considered significant when P ≤ 0.05.

### Consent

Written informed consent was obtained from the patient for the publication of this report and any accompanying images.

## Abbreviations

5-FU: 5 Fluourouracil; HBV: Hepatitis B virus; HCV: Hepatitis C virus; HCC: Hepatocellular carcinoma; NM: Nitrocellulose membrane; miRs: MicroRNAs; TUNEL: Terminal deoxynucleotidyl transferase dUTP nick end labeling; DAPI: 4′,6-Diamidino-2-Phenylindole; PT: Peritumoral; RTK: Receptor tyrosine kinase; 3′UTR: 3′ untranslated region; uPA: Urokinase-type plasminogen activator.

## Competing interests

The authors declare that they have no competing interests.

## Authors’ contribution

AS contributed intellectually toward the design, implementation and interpretation of the results and wrote the manuscript; IC performed the study of combined treatments of HCC cells with miRNAs and sorafenib and contributed to implementation and interpretation of the results; EA performed the expression study of miR193a in human HCC and contributed to the interpretation of the results. AS, IC, AB and IG performed the experimental validation of miR-193a in HCC cells. CS contributed to statistical analysis. NP and SB contributed to the discussion of the results; GDP outlined the experimental design of the study, contributed to the discussion of the results and revised the manuscript. All authors read and approved the final manuscript.

## Supplementary Material

Additional file 1: Table S1 Clinical and phatological characteristics of the studied population. Click here for file

Additional file 2 **miR-193a expression level detected by real-time PCR in tissues from biopsy specimens from patients affected by HCC.** The graph indicates the R (RQHCC/RQPT) corresponding to the human sample tested. The histograms are ordinated by increasing R. The background disease are also indicated (LC, liver cirrhosis; O, other background disease i.e., B/C viral hepatitis, steatosis). The R values and the case number (LV) are listed under the graph. Click here for file

Additional file 3 **Normal distribution of the R values (RQHCC/RQPT) of miR-193a detected by real-time PCR in tissues from biopsy specimens from patients affected by HCC.** The black curve indicates the normal distribution of R in all cases tested; the dashed black and grey curves refer to the HCC samples with, respectively, the presence or absence of liver cirrhosis as background disease. Click here for file

Additional file 4 **miR-193a expression level detected by real-time PCR in tissues from biopsy specimens from patients affected by HCC with the absence (A) or presence (B) of liver cirrhosis as background disease.** The graph indicates the R (RQHCC/RQPT) corresponding to the human sample tested. The histograms are ordinated by increasing R. The background diseases are also indicated (LC, liver cirrhosis; O, other background disease i.e., B/C viral hepatitis, steatosis). The R values and the case number (LV) are listed under the graph. Click here for file

Additional file 5 **Non cirrhotic HCCs N=14.** Stratification of the non-cirrhotic HCCs on the basis of the type of hepatitis virus infection. HBV (n=5); HCV (n=5); HBV/HCV (n=0); -/- (n=2). The miR-193a is down-modulated in the HCC patients with and without cirrhosis subdivided on the basis of the HCV virus infection (n=15). The mean R value was 0.604±0,14 which was significantly different from the expected value=1, p=0.0167. Click here for file

Additional file 6 **Loading controls relative to Figures **2**A, B, A and Figure **3**B.** Coomassie staining of proteins loaded on polyacrylamide gels 8% (panel A, left) and 4-12% pre-cast gels (panel A, right and panel B). The staining highlights constant amounts of total proteins loaded for each gel. Click here for file
