# Supplementary material for: Effects of miR-193a and sorafenib on hepatocellular carcinoma cells
Source: Mol Cancer. 2013 Dec 13;12:162. doi: 10.1186/1476-4598-12-162 (PMC4029516; doi:10.1186/1476-4598-12-162)
Supplement: Additional file 3 — Normal distribution of the R values (RQHCC/RQPT) of miR-193a detected by real-time PCR in tissues from biopsy specimens from patients affected by HCC. The black curve indicates the normal distribution of R in all cases tested; the dashed black and grey curves refer to the HCC samples with, respectively, the presence or absence of liver cirrhosis as background disease. [file 1476-4598-12-162-S3.ppt]

## Slide 1
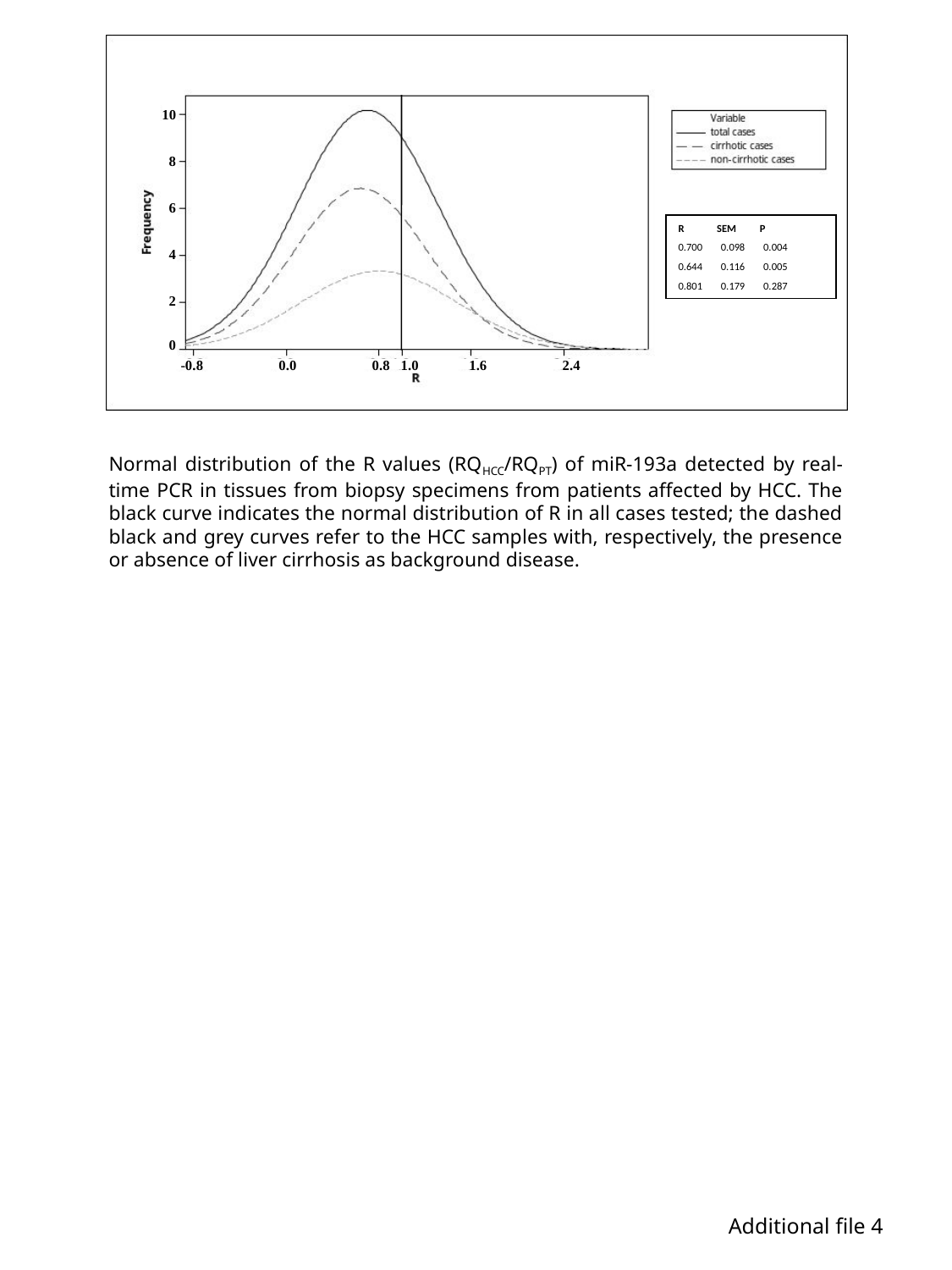

R SEM P
0.700 0.098 0.004
0.644 0.116 0.005
0.801 0.179 0.287
-0.8 0.0 0.8 1.0 1.6 2.4
10
 8
 6
 4
 2
 0
Normal distribution of the R values (RQHCC/RQPT) of miR-193a detected by real-time PCR in tissues from biopsy specimens from patients affected by HCC. The black curve indicates the normal distribution of R in all cases tested; the dashed black and grey curves refer to the HCC samples with, respectively, the presence or absence of liver cirrhosis as background disease.
Additional file 4
