# Supplementary material for: Effects of miR-193a and sorafenib on hepatocellular carcinoma cells
Source: Mol Cancer. 2013 Dec 13;12:162. doi: 10.1186/1476-4598-12-162 (PMC4029516; doi:10.1186/1476-4598-12-162)
Supplement: Additional file 4 — miR-193a expression level detected by real-time PCR in tissues from biopsy specimens from patients affected by HCC with the absence (A) or presence (B) of liver cirrhosis as background disease. The graph indicates the R (RQHCC/RQPT) corresponding to the human sample tested. The histograms are ordinated by increasing R. The background diseases are also indicated (LC, liver cirrhosis; O, other background disease i.e., B/C viral hepatitis, steatosis). The R values and the case number (LV) are listed under the graph. [file 1476-4598-12-162-S4.ppt]

## Slide 1
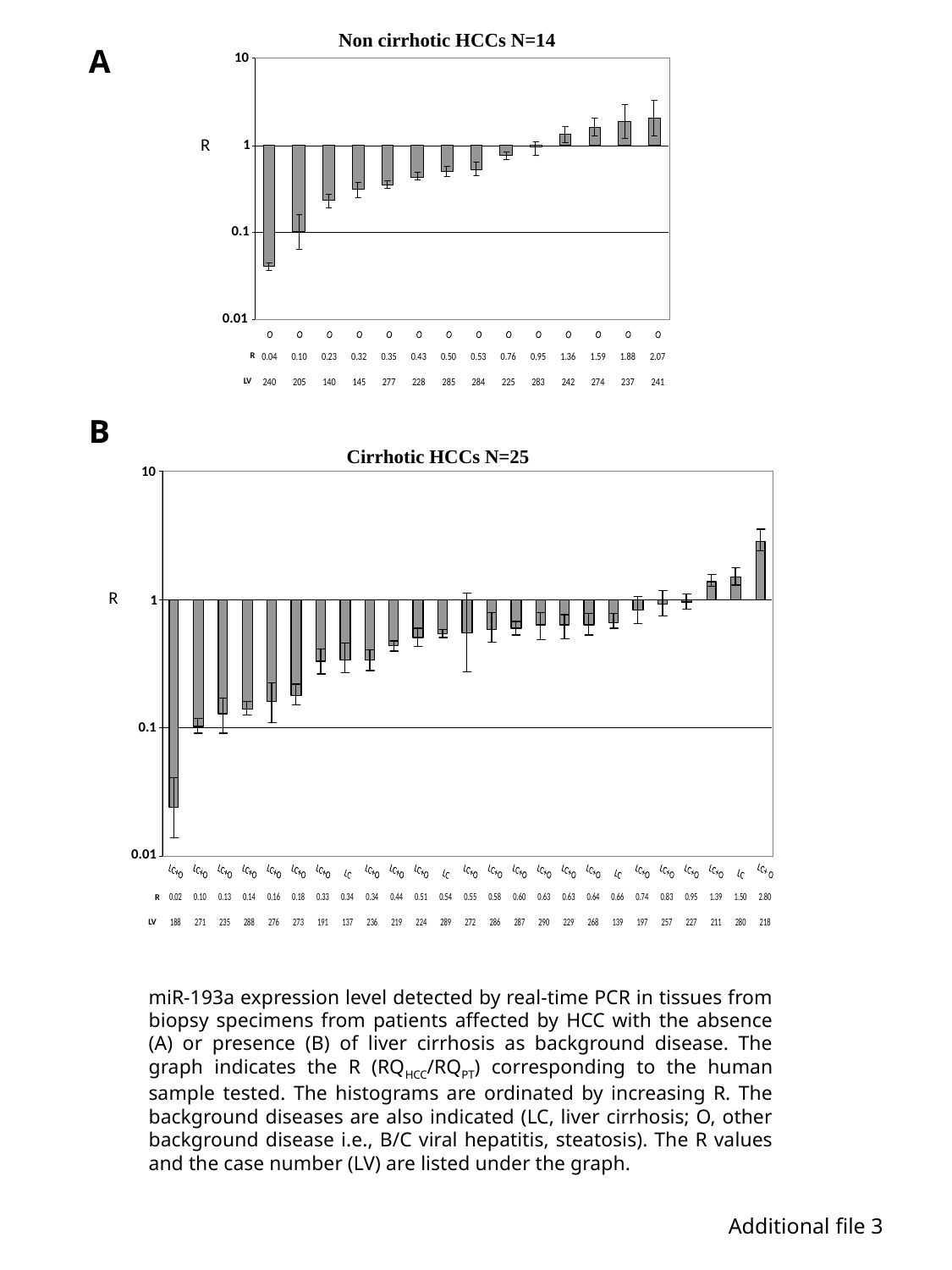

Non cirrhotic HCCs N=14
A
10
R
1
0.1
0.01
R
LV
B
Cirrhotic HCCs N=25
10
R
1
0.1
0.01
R
LV
miR-193a expression level detected by real-time PCR in tissues from biopsy specimens from patients affected by HCC with the absence (A) or presence (B) of liver cirrhosis as background disease. The graph indicates the R (RQHCC/RQPT) corresponding to the human sample tested. The histograms are ordinated by increasing R. The background diseases are also indicated (LC, liver cirrhosis; O, other background disease i.e., B/C viral hepatitis, steatosis). The R values and the case number (LV) are listed under the graph.
Additional file 3
