# Supplementary material for: Effects of miR-193a and sorafenib on hepatocellular carcinoma cells
Source: Mol Cancer. 2013 Dec 13;12:162. doi: 10.1186/1476-4598-12-162 (PMC4029516; doi:10.1186/1476-4598-12-162)
Supplement: Additional file 5 — Non cirrhotic HCCs N=14. Stratification of the non-cirrhotic HCCs on the basis of the type of hepatitis virus infection. HBV (n=5); HCV (n=5); HBV/HCV (n=0); -/- (n=2). The miR-193a is down-modulated in the HCC patients with and without cirrhosis subdivided on the basis of the HCV virus infection (n=15). The mean R value was 0.604±0,14 which was significantly different from the expected value=1, p=0.0167. [file 1476-4598-12-162-S5.ppt]

## Slide 1
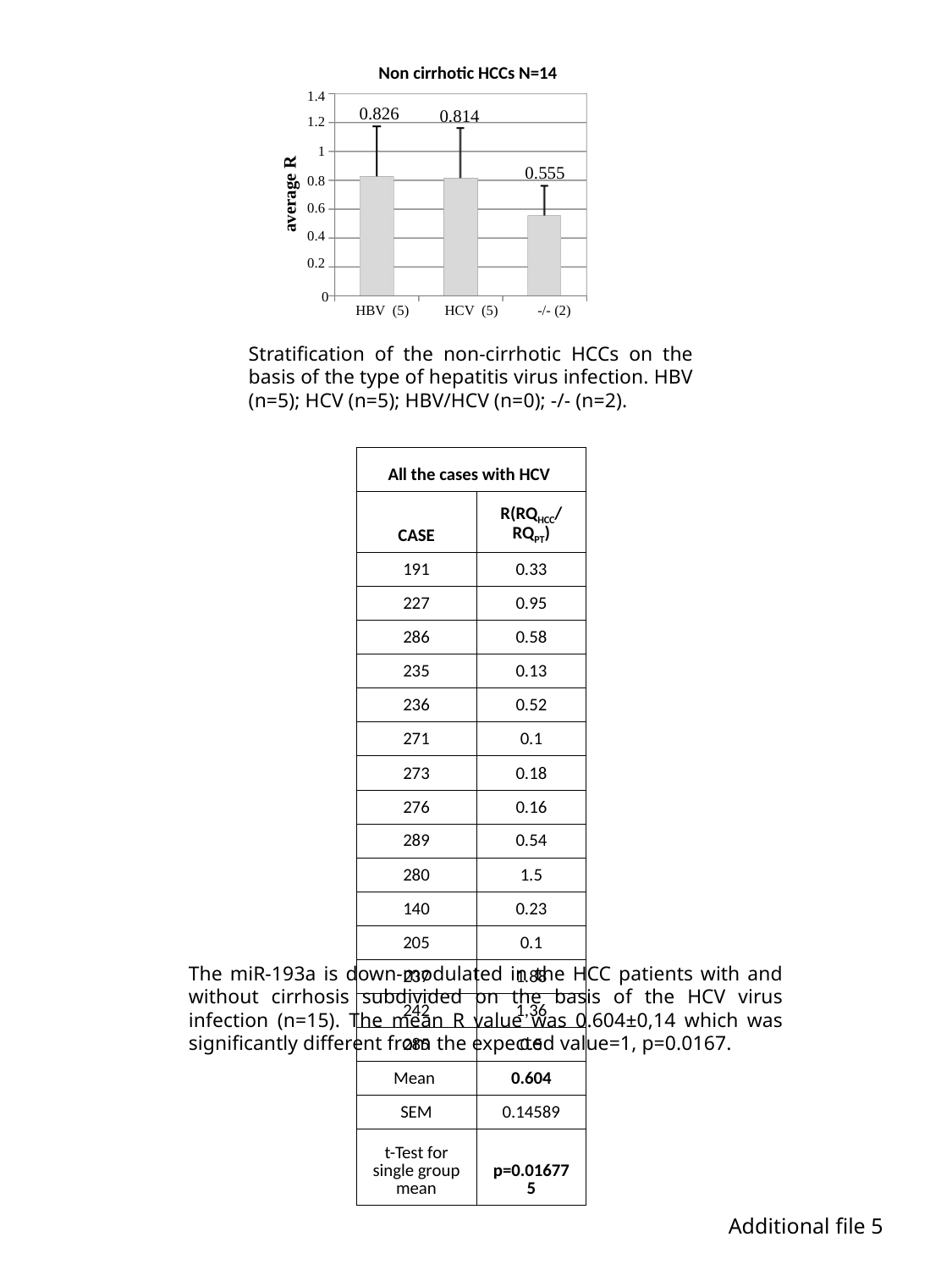

Non cirrhotic HCCs N=14
1.4
1.2
 1
0.8
0.6
0.4
0.2
 0
0.826
0.814
0.555
average R
 HBV (5) HCV (5) -/- (2)
Stratification of the non-cirrhotic HCCs on the basis of the type of hepatitis virus infection. HBV (n=5); HCV (n=5); HBV/HCV (n=0); -/- (n=2).
| All the cases with HCV | |
| --- | --- |
| CASE | R(RQHCC/RQPT) |
| 191 | 0.33 |
| 227 | 0.95 |
| 286 | 0.58 |
| 235 | 0.13 |
| 236 | 0.52 |
| 271 | 0.1 |
| 273 | 0.18 |
| 276 | 0.16 |
| 289 | 0.54 |
| 280 | 1.5 |
| 140 | 0.23 |
| 205 | 0.1 |
| 237 | 1.88 |
| 242 | 1,36 |
| 285 | 0.5 |
| Mean | 0.604 |
| SEM | 0.14589 |
| t-Test for single group mean | p=0.016775 |
The miR-193a is down-modulated in the HCC patients with and without cirrhosis subdivided on the basis of the HCV virus infection (n=15). The mean R value was 0.604±0,14 which was significantly different from the expected value=1, p=0.0167.
Additional file 5
