# Supplementary material for: Effects of miR-193a and sorafenib on hepatocellular carcinoma cells
Source: Mol Cancer. 2013 Dec 13;12:162. doi: 10.1186/1476-4598-12-162 (PMC4029516; doi:10.1186/1476-4598-12-162)
Supplement: Additional file 6 — Loading controls relative to Figures 2A, B, A and Figure 3B. Coomassie staining of proteins loaded on polyacrylamide gels 8% (panel A, left) and 4-12% pre-cast gels (panel A, right and panel B). The staining highlights constant amounts of total proteins loaded for each gel. [file 1476-4598-12-162-S6.ppt]

## Slide 1
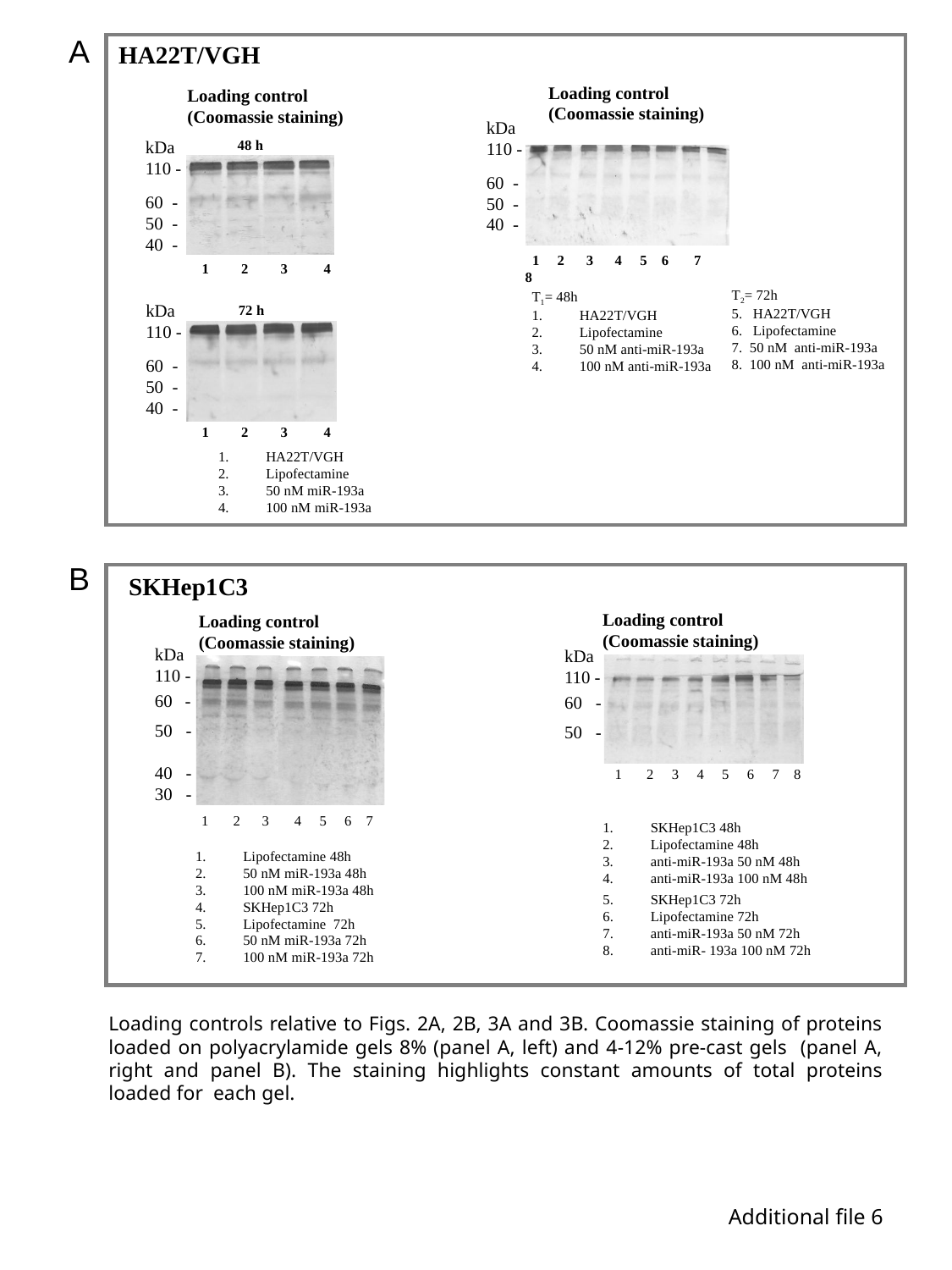

A
HA22T/VGH
Loading control
(Coomassie staining)
Loading control
(Coomassie staining)
kDa
110 -
60 -
50 -
40 -
kDa
110 -
60 -
50 -
40 -
48 h
 1 2 3 4 5 6 7 8
 1 2 3 4
T2= 72h
5. HA22T/VGH
6. Lipofectamine
7. 50 nM anti-miR-193a
8. 100 nM anti-miR-193a
T1= 48h
HA22T/VGH
Lipofectamine
50 nM anti-miR-193a
100 nM anti-miR-193a
kDa
110 -
60 -
50 -
40 -
72 h
HA22T/VGH
Lipofectamine
50 nM miR-193a
100 nM miR-193a
 1 2 3 4
B
SKHep1C3
Loading control (Coomassie staining)
Loading control (Coomassie staining)
kDa
110 -
-
50 -
40 -
30 -
kDa
110 -
60 -
50 -
2 3 4 5 6 7 8
SKHep1C3 48h
Lipofectamine 48h
anti-miR-193a 50 nM 48h
anti-miR-193a 100 nM 48h
2 3 4 5 6 7
Lipofectamine 48h
50 nM miR-193a 48h
100 nM miR-193a 48h
SKHep1C3 72h
Lipofectamine 72h
50 nM miR-193a 72h
100 nM miR-193a 72h
5. 	SKHep1C3 72h
6. 	Lipofectamine 72h
7. 	anti-miR-193a 50 nM 72h
8. 	anti-miR- 193a 100 nM 72h
Loading controls relative to Figs. 2A, 2B, 3A and 3B. Coomassie staining of proteins loaded on polyacrylamide gels 8% (panel A, left) and 4-12% pre-cast gels (panel A, right and panel B). The staining highlights constant amounts of total proteins loaded for each gel.
Additional file 6
